# More Cancer Research

**Published:** 1905-01-14

**Authors:** 


### MORE CANCER RESEARCH.

FOLLOWING up the observations of Farmer, Moore, and Walker, Mr. V. Chastel de Boinville, assistant director, Liverpool Cancer Research, has been making a study of the nature of the follicle cells of the mammalian ovary, and especially of the mitotic changes which occur in these cells. The follicle cells are those which form the zona glomerulosa and the discus proligerus of the Graafian follicle. Suspicion has recently fallen upon these cells as possibly playing some part in the growth of malignant tumours, and this suspicion is rather increased than diminished by Mr. de Boinville's observations. He states that after a careful investigation, involving the examination of many hundreds of nuclei belonging to the follicle cells of the mammalian ovary, he has been led to believe that the type of mitotic division prevailing in these cells is heterotype, that is to say, the mitotic figures are characterised by a reduced number of chromosomes. These chromosomes are of a coarse nature as compared with those of the cells of the rest of the body, and they closely resemble those that occur in cancer and sarcoma, while the general character and the method of division are similar to what obtains in the parent cells of the ovum or spermatozoon.

